# *Haplodontiumaltunense* (Bryaceae, Bryopsida), a new moss species from Northwest China

**DOI:** 10.3897/phytokeys.183.71642

**Published:** 2021-10-08

**Authors:** Xiao-Rui Wang, Min Li*, John R. Spence, Jian-Cheng Zhao, Sulayman Mamtimin

**Affiliations:** 1 College of Resources and Environmental Science, Shijiazhuang University, Shijiazhuang 050035, China Shijiazhuang University Shijiazhuang China; 2 College of Life Science, Hebei Normal University, Shijiazhuang 050024, China Hebei Normal University Shijiazhuang China; 3 California Academy of Sciences, Department of Botany, 55 Music Concourse Drive, Golden Gate Park, CA 94118-4503, San Francisco, USA California Academy of Sciences San Francisco United States of America; 4 College of Life Science and Technology, Xinjiang University, Urumqi 830046, China Xinjiang University Urumqi China

**Keywords:** Altun Mountain, *Bryum*, ITS, *Plagiobryoides*, *Ptychostomum*

## Abstract

*Haplodontiumaltunense* X.R.Wang & S.Mamtimin, a new moss species of the family Bryaceae from Xinjiang Uygur Autonomous Region, China is described and illustrated. Genetic analysis based on ITS sequences shows that this species is a member of the Bryaceae and in the same clade as *Anomobryum*. Particularly distinctive features of the new species include: double peristome; the exostome has raised and membranous chomata with united lamellae between two teeth proximally; the endostome is poorly developed and all the endostomial material tightly adherent to the exostome.

## Introduction

*Haplodontium* Hampe, classified in the family Bryaceae, has been confused with the genus *Mielichhoferia* Hornsch. Shaw and Crum (1984) transferred all species in the genus to *Mielichhoferia* because of the similar peristome. Genetic research (Cox et al. 2000; Goffinet et al. 2001; Pedersen et al. 2003, 2007; Holyoak and Pedersen 2007) has clearly shown that some species originally in *Haplodontium* are nested within Bryaceae. Spence (2005) resurrected the genus *Haplodontium* and transferred two North American species of *Mielichhoferia* to *Haplodontium*. In addition, the genus *Mielichhoferia* has been accommodated in the family Mielichhoferiaceae (Shaw 2009). Pedersen et al. (2007) showed that *Haplodontium*, *Acidodontium* Schwägr. and *Anomobryum* Schimp. are sister taxa and *Haplodontium* should be included in the family Bryaceae.

## Materials and methods

### Morphological observations

Microscopic examination was carried out using traditional methods. The collections of *Haplodontium* and relevant species of Bryaceae in the herbarium of Hebei Normal University (HBNU), Institute of Applied Ecology, Chinese Academy of Sciences (IFP), Kunming Institute of Botany, Chinese Academy of Sciences (KUN), Institute of Botany, the Chinese Academy of Sciences (PE) and Xinjiang University (XJU) were examined.

Authors observed the plants under the dissecting microscope and examined the leaves, capsules and peristome under the compound light microscope and scanning electron microscope. Light micrographs were photographed using a Nikon E-800 microscope with a Nikon DXM1200F digital camera. The peristome and spores were mounted on double sided sticky tape on aluminium stubs, gold-coated and viewed using a Hitachi S-4800 field emission SEM. All line drawings were made using the drawing tube attachments of these optical microscopes.

### Phylogenetic analyses

Twenty-one samples were used for the analyses (Table 1). To evaluate the systematic position of *Haplodontiumaltunense*, 20 representatives of allied genera in the family Bryaceae, including *Anomobryum*, *Bryum*, *Gemmabryum*, *Plagiobryum* and *Ptychostomum*, were also sampled as part of the ingroup (Cox et al. 2000; Goffinet et al. 2001; Pedersen et al. 2003, 2007; Kato et al. 2013). *Bryumargenteum* was selected as an outgroup. In addition to 10 sequences from GenBank, 11 sequences newly produced for the present study were included.

**Table 1.** Voucher information and GenBank accession numbers of taxa used in the phylogenetic analyses.

Taxon	Voucher (Herbarium)	Origin	GenBank No.	Source
*Anomobryum auratum 1*	L.B. Li 20073626 (HBNU)	China	MZ470251	This study
*Anomobryum auratum 2*	L.B. Li 20073628 (HBNU)	China	MZ470252	This study
*Anomobryumjulaceum*	L.B. Li 20072925 (HBNU)	China	FJ796895	Wang et al. 2011
*Bryumargenteum*	X.R. Wang 20150512031 (HBNU)	China	MZ470253	This study
*Bryumparadoxum*	J.C. Zhao 0610067 (HBNU)	China	EU878207	Wang et al. 2011
*Bryumrecurvulum*	W.Q. Li 040900 (HBNU)	China	EU878217	Wang et al. 2011
*Gemmabryumcaespiticium*	X.R. Wang 20156001 (HBNU)	China	MZ470254	This study
*Haplodontiumaltunense*	S. Mamtimin 16752 (XJU)	China	MZ470255	This study
*Plagiobryumzierii*	W.Q. Li 000514 (HBNU)	China	EU878219	Wang et al. 2011
*Ptychostomumarcticum*	S. Mamtimin 15457 (HBNU)	China	MZ470256	This study
*Ptychostomumbimum*	L. Hedenas B90015 (S)	Sweden	DQ381780	Holyoak and Hedenas 2006
*Ptychostomumcernuum*	YL Niu 110002 (HBNU)	China	MZ470257	This study
*Ptychostomuminclinatum*	N. Cao 20050085 (HBNU)	China	EU878227	Wang et al. 2011
*Ptychostomumlonchocaulon*	N. Cao 20050187 (HBNU)	China	FJ796878	Wang et al. 2011
*Ptychostomumneodamense*	L. Hedenas B65900 (S)	Sweden	DQ381772	Holyoak and Hedenas 2006
*Ptychostomumpallens*	M.X. Xiao 20091246 (HBNU)	China	MZ470258	This study
*Ptychostomumpallescens*	S. Mamtimin 15265 (HBNU)	China	MZ470259	This study
*Ptychostomumpendulum*	J.C. Zhao 20060463 (HBNU)	China	FJ796811	Wang et al. 2011
*Ptychostomumpseudotriquetrum*	D.T. Holyoak B90021 (S)	Ireland	DQ381774	Holyoak and Hedenas 2006
*Ptychostomumpurpurascens*	Y.L. Niu 110045 (HBNU)	China	MZ470260	This study
*Ptychostomumturbinatum*	S. Mamtimin 15095 (HBNU)	China	MZ470261	This study

Genomic DNA was extracted from freshly collected and silica gel-dried plants using a Plant Genomic DNA Kit (TIANGEN Biotech (Beijing) Co., Ltd.) according to the manufacturer’s protocol. One nuclear marker ITS was chosen. The following primers were used to amplify the marker: ‘18SF’ and ‘26SR’ for the ITS region, or sometimes ‘18SF’ and ‘5.8SR’ for ITS1, and ‘5.8SF’ and ‘26SR’ for ITS2 (Hartmann et al. 2006). PCR cycles used an initial denaturation step of 3 minutes at 95 °C, followed by 35 cycles of 30 seconds at 95 °C, 30 seconds at 50 °C, 90 seconds at 72 °C, and a final elongation of 5 minutes at 72 °C. PCR products were purified with a Gel Extraction Kit (Cwbio, Shanghai, China) following the instruction manual. These purified PCR products were sequenced by Life Technologies Inc., China (http://www.lifetechnologies.com).

Sequence chromatograms were compiled using SeqMan II (DNASTAR Inc., Madison, WI, USA) and then aligned manually in PhyDE 0.9971 (Müller et al. 2010). Regions of partially incomplete data at the beginnings and ends of sequences were identified and excluded from subsequent analyses. Gaps were treated as missing data. The aligned ITS dataset was composed of 1213 bp.

The maximum likelihood (ML) method was performed using RAxML v.8.2.12 on the CIPRES Science Gateway (http://www.phylo.org/), and inferred under the default settings (Stamatakis 2014). The fast bootstrap option was used with 1000 replicates. TreeGraph 2 (Stöver and Müller 2010) was used to summarize the topologies and support values from the analyses.

## Results

### Taxonomic treatment

#### Haplodontium altunense

Taxon classificationPlantaeBryalesBryaceae

X.R.Wang & S.Mamtimin sp. nov.

BEE0DFCF-2586-538E-A194-07DB46AC2158

Figs 1 , 2 , 3

##### Type.

China. Xinjiang, Ruoqiang County, Altun Mountain National Nature Reserve, 37°0.42'N, 88°36.35'E, 4290 m a.s.l., 22 July 2011, *S Mamtimin 16752* (***holotype***: HBNU!; ***isotype***: XJU!).

**Figure 1.**
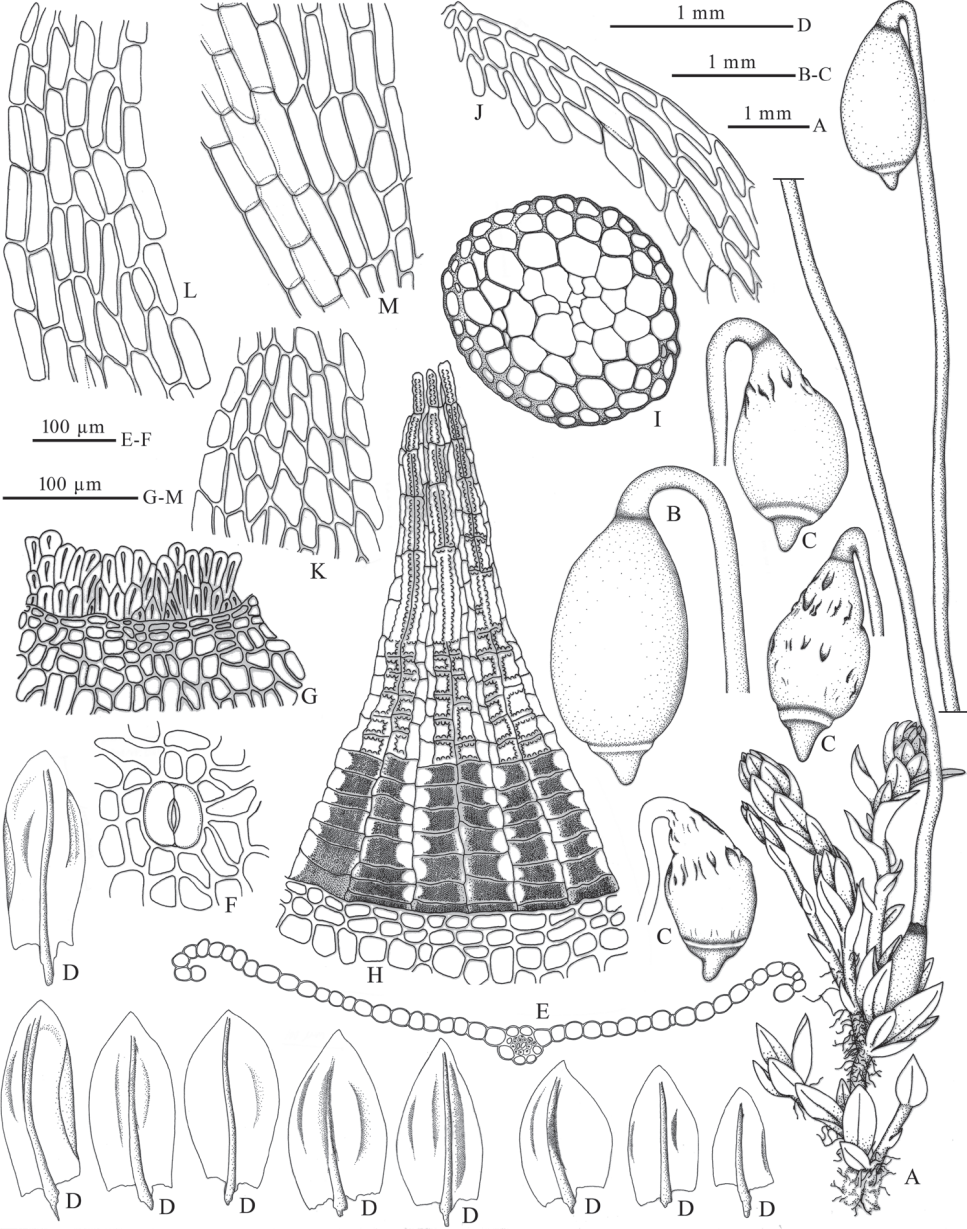
*Haplodontiumaltunense***A** plant (wet) **B** capsule (wet) **C** capsules (dry) **D** leaves **E** transverse section of leaf **F** superficial stoma **G** annulus growing on the capsule mouth **H** dorsal views of peristome **I** transverse section of stem **J** apical laminal cells and margin **K** apical laminal cells **L** median laminal cells **M** basal laminal cells. Drawn by Xiaorui Wang from the holotype (HBNU!).

##### Diagnosis.

Particularly distinctive features of the new species including: double peristome; the exostome has raised and membranous chomata with united lamellae between two teeth proximally; the endostome is poorly developed and all the endostomial material tightly adherent to the exostome.

**Figure 2.**
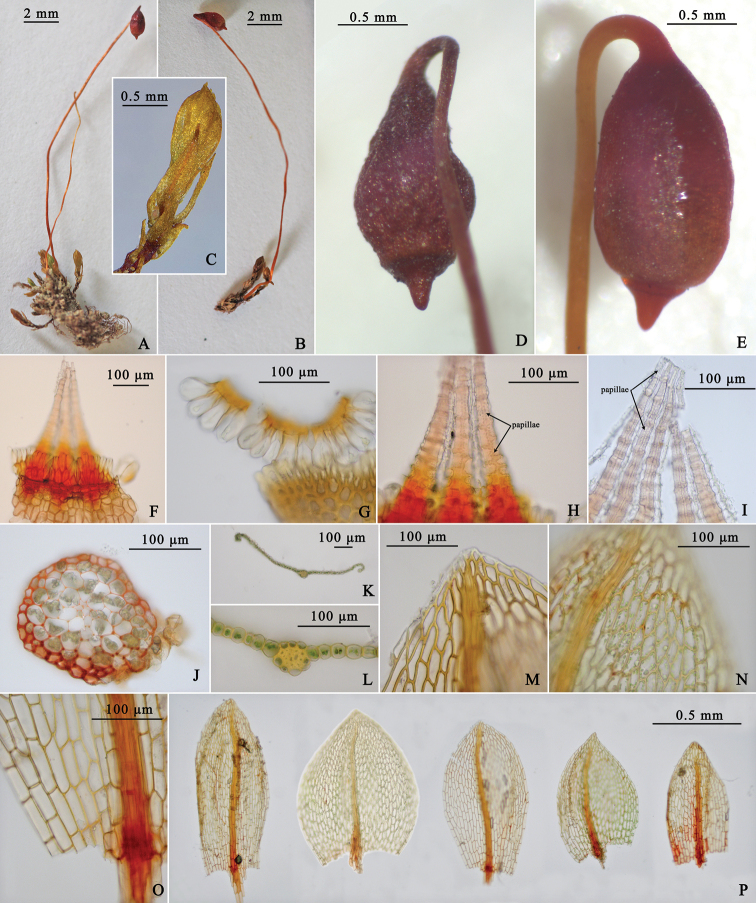
Light micrographs of *Haplodontiumaltunense***A–C** plants (dry) **D** Capsule (dry) **E** capsule (wet) **F** annulus growing on the capsule mouth **G** annulus falling off the capsule mouth **H** dorsal views of median peristome showing the large papillae along the horizontal and median vertical lines **I** dorsal views of distal peristome showing adherent endostomial material and exostome teeth **J** transverse section of stem **K** transverse section of midleaf **L** transverse section of costa **M** leaf apex **N** median laminal cells **O** basal laminal cells **P** leaves. Photographed by Xiaorui Wang from the holotype (HBNU!).

##### Description.

Plants small, soft and dull, brown-green. Stems short, 2.5–6 mm high, weakly julaceous, branched, circular or pentagonal circular in transverse section with small and thick-walled peripheral cells surrounding 2–3 layers gradually larger and thin-walled cortical cells, central strand weakly developed. Leaves imbricate when dry, erect when moist, enlarged towards stem apex, ovate to broadly ovate, concave, 0.5–1.1×0.3–0.7 mm; base not decurrent; margins plane or recurved medially, 1-stratose, limbidium absent, smooth or finely serrulate distally; apex broadly acute; costae not reaching apex, guide cells weakly developed, 2–4 in one layer in costal transverse section, ventral and dorsal stereid bands present; alar cells not differentiated from juxtacostal cells; laminal cells lax; distal laminal cells rhomboidal, 30–44×11–21 μm, with slightly thickened walls; medial laminal cells long rhomboidal to rectangular, 37–69×12–20 μm, somewhat narrower in 2 or 3 rows toward the margins but not forming a distinct border; proximal laminal cells long rectangular, 37–56×20–28 μm. Dioicous(?). Perigonia not seen. Perichaetia at the end of short, inconspicuous stems, appearing laterally because of well-developed innovations; perichaetial leaves larger than vegetative leaves. Setae single, light brown, 15–19 mm long. Capsules nutant and symmetric, reddish brown, obovoid, 1.5–2 mm, neck short and indistinct, mouth small, stomata abundant in the neck, superficial; opercula long-conic with short rostrate; annulus present, consisting of two rows of cells, revoluble and cells with slit-like lumen; peristome double, exostome inserted below the mouth, teeth lanceolate, red-brown and pored, raised and membranous chomata with united lamellae between two teeth proximally, pale yellow to hyaline and largely papillose along horizontal and median vertical lines distally; endostome poorly developed, basal membrane smooth, segments and cilia rudimentary, all the endostomial material strongly adhere to the exostome. Spores spherical, 20–22 μm in diameter, minutely papillose.

**Figure 3.**
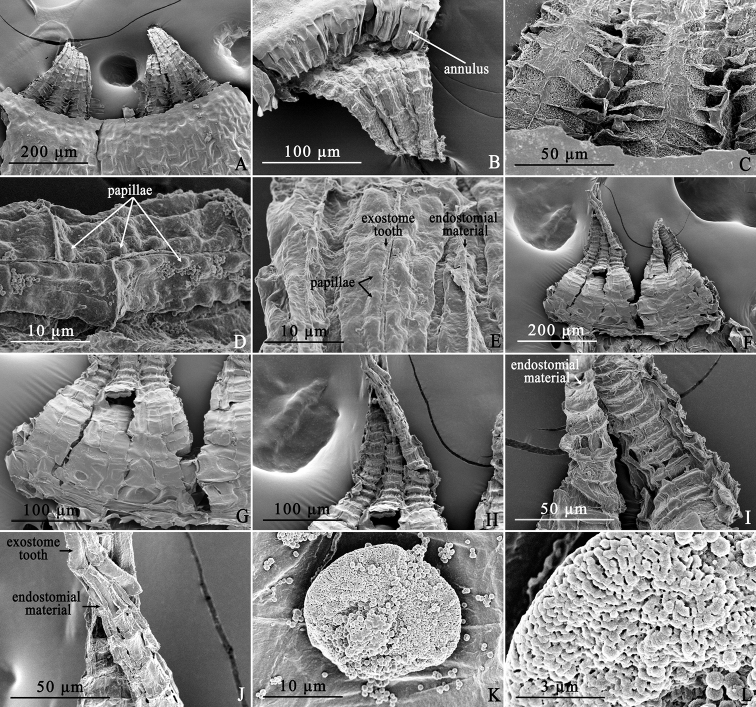
Scanning electron micrographs of *Haplodontiumaltunense***A** dorsal views of peristome **B** dorsal views of peristome with annulus **C** dorsal surface of exostome teeth proximally showing the pores and the raised and membranous chomata horizontal lines with united lamellae between two teeth **D** dorsal surface of exostome teeth distally showing the large papillae along the median vertical lines **E** dorsal surface of exostome teeth distally showing the endostomial material adherent to the teeth **F** ventral views of peristome **G** ventral surface of peristome proximally showing the smooth endostome basal membrane **H** ventral views of peristome distally **I, J** ventral surface of peristome medially and distally showing the endostomial material adherent to the teeth **K** spore **L** exine ornamentation of spore. Photographed by Xiaorui Wang from the holotype (HBNU!).

##### Etymology.

The specific epithet altunense refers to the type locality in Altun Mountain National Nature Reserve in the Northwestern China.

##### Distribution and habitat.

China (Xinjiang). Only known from the type locality, on soil substrates at 4290 m in the Altun Mountain National Nature Reserve. The population grows in a dry, cold, and windy habitat with intense evaporation. The companion species include some xerophytic mosses of the family Pottiaceae.

##### Chinese name.

阿尔金拟缺齿藓 (ā ěr jīn nĭ quē chĭ xĭan)

### Phylogenetic analyses

**Figure 4.**
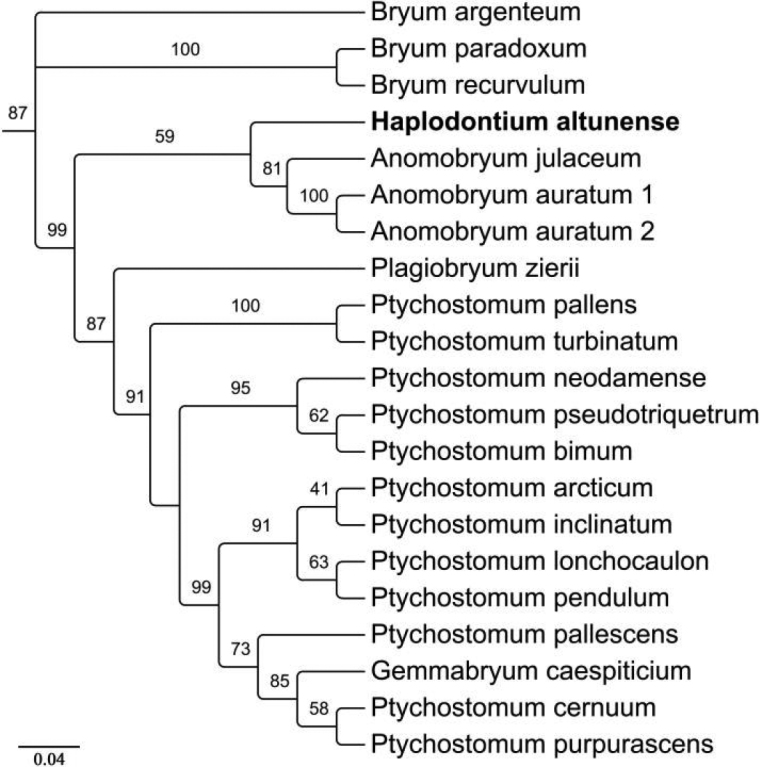
Phylogenetic relationships of *Haplodontiumaltunense* sp. nov. from ITS rDNA with related genera in Bryaceae based on Maximum Likelihood analysis. Support values are given above branches. *Bryumargenteum* was served as an outgroup.

The present phylogenetic analysis, based on the nuclear ribosomal internal transcribed spacer region ITS1–5.8S–ITS2 (hereafter, ITS) region, included 20 species from six genera, as well as *Bryumargenteum* Hedw. as outgroup (Fig. 4). The only sample of *Haplodontiumaltunense* is sister to the *Anomobryum* clade (81 MLBS), which is monophyletic with three members. Twelve samples of *Ptychostomum* Hornsch. formed a monophyletic clade (91 MLBS), in which *Gemmabryumcaespiticium* (Hedw.) J.R. Spence was nested. *Plagiobryumzierii* (Hedw.) Lindb. is closely related to the *Ptychostomum* clade. The *Bryum* Hedw. clade (100 MLBS) with three species were basal to the main clades.

## Discussion

Pending a careful examination of Bryaceae in the Altun Mountains, we discovered an unusual collection with distinctive morphological features of the peristome: endostome poorly developed and strongly adherent to the exostome. We thought this collection could belong to the genus *Synthetodontium* Cardot at first sight based on its fused peristome. Phylogenetic analysis showed that this collection was nested into Bryaceae clade. It is genetically distinct from the closely related *Anomobryum* group (Fig. 4). Further examination revealed that it has morphological characters of *Haplodontium* such as stems nearly julaceous, leaf margins entire, distal and medial laminal cells laxly rhomboidal to rectangular. However, the collection is distinguished from the other previously recognized species in the region by its double peristome, and raised and membranous chomata with united lamellae between two exostome teeth proximally. We thus describe it as a new species of the genus *Haplodontium*.

The gametophytes of *Haplodontium* species are similar to those of *Plagiobryum* and *Plagiobryoides* J.R.Spence (Spence 2015). Genetic research has shown that the type species of *Haplodontium* is closely related to *Acidodontium* and *Anomobryum* (Cox and Hedderson 2003; Pedersen et al. 2007). Peristome reduction is common and complex in *Haplodontium*, from double to single to absent (Spence 2005).

The gametophyte characters of *Haplodontiumaltunense* are similar to *Plagiobryoidesbrachyneura* (Kindberg) J.R.Spence (Spence 2015). At the same time, both species have double, reduced and fused peristome. In *P.brachyneura*, setae are red-brown and 5–15 mm long, capsules are inclined to nutant, elongate-pyriform and 2–4 mm long with elongate-neck, opercula are weakly convex, exostome teeth are short and irregular in shape. However, those characters of *H.altunense* are as follows: setae are light brown and 15–19 mm long, capsules are nutant, pyriform to obovoid and 1.5–2 mm long with indistinct short-neck, opercula are long-conic with short rostrate, exostome teeth are regular long lanceolate.

*Haplodontiumaltunense* is also similar to *Ptychostomumpendulum* Hornschuch (Spence 2015) (≡*Bryumalgovicum* Sendtner ex Müller Hal. (Li 2006; Zhang et al. 2007) in that the endostome adheres to the exostome teeth. While the former species differ from the latter one in length of stems (2.5–6 mm vs. 5–20 mm), leaf apex (broadly acute or obtuse vs. acuminate), leaf margin (indistinct bordered vs. strong limbidium), costae (ending at or near the apex vs. long-excurrent in denticulate awn), exostome teeth (united at the base, large papillose above vs. separate, smooth above), endostome (segments reduced vs. segments with ovate perforations).

Wang et al. (2017) reported one new species, *Haplodontiumzangii* X.R.Wang & J.C.Zhao, from Tibet, China and transferred two Chinese species in *Mielichhoferia* to *Haplodontium* as new combinations: *H.himalayanum* (Mitt.) X.R.Wang & J.C.Zhao and *H.sinensis* (Dix.) X.R.Wang & J.C.Zhao. The most significant difference among the four species of *Haplodontium* in China is that the first three species all have single peristome, while *H.altunense* has double and fused peristome.

### Key to the *Haplodontium* species in China

**Table:** 

1	Peristome double, exostome teeth lanceolate, raised and membranous chomata with united lamellae between two teeth proximally, endostome reduced, all the endostomial material strongly adhere to the exostome	***H.altunense***
–	Peristome single, exostomial	**2**
2	Leaves lanceolate; costae excurrent, ending in long denticulate awns, awns 140–310 μm long	***H.himalayanum***
–	Leaves ovate to oblong-ovate; costae subpercurrent to ending in short awns, awns 0–130 μm long	**3**
3	Leaf apex cucullate; capsules pyriform; exostome teeth regularly lanceolate, not perforate, vertically striped on dorsal surface, smooth on ventral surface	***H.sinensis***
–	Leaf apex plane; capsules subglobose to short pyriform; exostome teeth irregularly linear-lanceolate, sometimes perforate, smooth or finely papillose	***H.zangii***

## Supplementary Material

XML Treatment for Haplodontium altunense

## References

[B1] Cox CJ Hedderson TAJ (2003) Phylogenetic relationships within the moss family Bryaceae based on chloroplast DNA evidence. Journal of Bryology 25(1): 31–40. 10.1179/037366803125002635

[B2] Cox CJ Goffinet B Newton AE Shaw AJ Hedderson TAJ (2000) Phylogenetic relationships among the diplolepideous-alternate mosses (Bryidae) inferred from nuclear and chloroplast DNA sequences. The Bryologist 103(2): 224–241. 10.1639/0007-2745(2000)103[0224:PRATDA]2.0.CO;2

[B3] Goffinet B Cox CJ Shaw AJ Hedderson TAJ (2001) The Bryophyta (mosses): Systematic and evolutionary inferences from an rps4 gene (cpDNA) phylogeny. Annals of Botany 87(2): 191–208. 10.1006/anbo.2000.1318 32050736

[B4] Hartmann FA Wilson R Gradstein SR Schneider H Heinrichs J (2006) Testing Hypotheses on Species Delimitations and Disjunctions in the Liverwort *Bryopteris* (Jungermanniopsida: Lejeuneaceae). International Journal of Plant Sciences 167(6): 1205–1214. 10.1086/508023

[B5] Holyoak DT Hedenäs L (2006) Morphological, ecological and molecular studies of the intergrading taxa *Bryumneodamense* and *B.pseudotriquetrum* (Bryopsida: Bryaceae). Journal of Bryology 28(4): 299–311. 10.1179/174328206X136304

[B6] Holyoak DT Pedersen N (2007) Conflicting molecular and morphological evidence of evolution within the Bryaceae (Bryopsida) and its implications for generic taxonomy. Journal of Bryology 29(2): 111–124. 10.1179/174328207X189198

[B7] Kato K Arikawa T Imura S Kanda H (2013) Molecular identification and phylogeny of an aquatic moss species in Antarctic lakes. Polar Biology 36(11): 1557–1568. 10.1007/s00300-013-1373-x

[B8] Li XJ (2006) Flora Bryophytorum Sinicorum (Vol. 4). Science Press, Beijing, 263 pp.

[B9] Müller K Quand D Mülle J Neinhuis C (2010) PhyDE: Phylogenetic Data Editor, ver. 0.9971, computer program. http://www.phyde.de [accessed 11.23.2010]

[B10] Pedersen N Cox CJ Hedenäs L (2003) Phylogeny of the moss family Bryaceae inferred from chloroplast DNA sequences and morphology. Systematic Botany 28(3): 471–482. https://www.jstor.org/stable/25063888

[B11] Pedersen N Holyoak DT Newton AE (2007) Systematics and morphological evolution within the moss family Bryaceae: A comparison between parsimony and Bayesian methods for reconstruction of ancestral character states. Molecular Phylogenetics and Evolution 43(3): 891–907. 10.1016/j.ympev.2006.10.018 17161629

[B12] Shaw AJ (2009) Mielichhoferiaceae. Bryophyte flora of North America, Provisional Publication (Version 1). Missouri Botanical Garden Press, St. Louis. http://www.mobot.org/plantscience/BFNA/V2/MielMielichhoferiaceae.htm [accessed 07.07.2014]

[B13] Shaw AJ Crum H (1984) Peristome homology in Mielichhoferia and a taxonomic account of North American species. The Journal of the Hattori Botanical Laboratory 57: 363–381.

[B14] Spence JR (2005) New genera and combinations in Bryaceae (Bryales, Musci) for North America. Phytologia 87: 15–28. 10.5962/bhl.part.4029

[B15] Spence JR (2015) Bryaceae. Flora of North America (Vol. 28). http://www.efloras.org/florataxon.aspx?flora_id=1&taxon_id=10129 [accessed 11.17.2015]

[B16] Stamatakis A (2014) RAxML version 8: A tool for phylogenetic analysis and post-analysis of large phylogenies. Bioinformatics (Oxford, England) 30(9): 1312–1313. 10.1093/bioinformatics/btu033 PMC3998144 24451623

[B17] Stöver BC Müller KF (2010) TreeGraph 2: Combining and Visualizing Evidence from Different Phylogenetic Analyses. BMC Bioinformatics 11(1): e7. 10.1186/1471-2105-11-7 PMC2806359 20051126

[B18] Wang CY Li DL Zhao JC (2011) New Evidence of phylogeny in Bryaceae (Musci) based on the ITS region. Bulletin of Botanical Research 31(6): 664–673. http://bbr.nefu.edu.cn/CN/10.7525/j.issn.1673-5102.2011.06.005

[B19] Wang XR Li M Zhao JC (2017) *Haplodontiumzangii* X.R.Wang & J.C.Zhao, sp. nov. (Bryaceae, Bryopsida) from Xizang, China. Journal of Bryology 39(3): 267–276. 10.1080/03736687.2017.1302149

[B20] Zhang DC Li XJ He S (2007) Bryaceae. In: Li XJ, Crosby MR, He S (Eds) Moss Flora of China. English Version (Vol. 4). Science Press, Beijing and Missouri Botanical Garden Press, St. Louis, 211 pp.

